# Evaluation of precision and trueness of intraoral scanners for newborns with cleft lip and palate: 3D analysis of digital models

**DOI:** 10.1186/s13005-026-00650-w

**Published:** 2026-09-09

**Authors:** Juliana-Theresa Schell, Bert Braumann, Teresa Kruse, Sarah Achterrath, Tamara N. Shamlian, Isabelle Graf

**Affiliations:** 1 Department of Orthodontics and Dentofacial Orthopedics, Medical Faculty and University Hospital of Cologne, Kerpener Str. 32, Cologne, 50931 Germany; 2 Private Practice, Fresno, CA USA

**Keywords:** Digitalization, Cleft lip and palate, Intraoral scanner, 3D printing

## Abstract

**Background:**

Children born with cleft lip and palate (CLP) require intensive interdisciplinary care from birth onward. Intraoral scanners (IOS) have emerged as a promising alternative for neonatal maxillary data acquisition. This study evaluated the precision and trueness of five commonly used IOS using a standardized landmark-based methodology complemented by full-surface deviation analysis, applied to neonatal maxillary models with increasing morphological complexity.

**Methods:**

Three neonatal plaster casts were digitized: An edentulous maxilla without cleft, a unilateral and a bilateral cleft maxilla. Ten pyramidal landmarks defining five transversal distances were constructed along the alveolar ridge. These resulting digital master models (DMM) were 3D-printed (Formlabs Form 2, 25 μm) and their dimensional fidelity independently verified by digital caliper (± 0.01 mm). Each printed model was scanned ten times by the same operator using Primescan, CS3600, Trios 3, iTero Element 2 and Medit i500. Linear trueness and precision were quantified in Rhinoceros 3D. Full-surface trueness was assessed in Geomagic Control 3D using a standardized alignment protocol. Statistical analysis was performed using the Kruskal–Wallis test and Mann–Whitney U post-hoc tests (*p* < 0.05).

**Results:**

Trueness varied between IOS and depended on the combination of device and cleft morphology. Mean signed linear deviations were minimal in the non-cleft model (all scanners within ± 0.03 mm) and reached − 0.147 mm for Medit in the bilateral cleft model, where Primescan and Trios remained essentially unbiased in the non-cleft and bilateral cleft models. Between-scanner differences were significant in both cleft models (*p* < 0.001). Intra-scanner reliability, an aspect of precision, ranged from ICC 0.832 to 1.000. Full-surface trueness differed significantly between scanners in all three models (*p* < 0.01). Mean RMS deviations remained below 0.07 mm for four of the five devices in every model, whereas iTero reached 0.224 mm in the bilateral cleft model. Compared with the non-cleft model, in-tolerance percentages decreased in both cleft models, most markedly in posterior and undercut regions.

**Conclusion:**

Under in-vitro conditions, all IOS digitized neonatal maxillary anatomy with high technical accuracy. Trueness and precision depended on the combination of device and cleft morphology rather than on morphological complexity alone, but all IOS captured the relevant morphological features. Clinical feasibility in neonates was not assessed and remains to be established in in-vivo studies.

**Clinical trial number:**

Not applicable.

**Supplementary Information:**

The online version contains supplementary material available at https://doi.org/10.1186/s13005-026-00650-w.

## Introduction

Children born with cleft lip and palate (CLP) require intensive interdisciplinary medical care from birth onward [1]. Although no standardized national or international treatment protocol exists, early therapeutic measures such as presurgical infant orthopedics (PSIO) have been shown to support maxillary growth and improve surgical conditions before lip and palate repair [2, 3]. PSIO typically begins within the first 48 h of life and involves the fabrication of a passive palatal plate to mold the maxillary segments and facilitate feeding and nasal symmetry [4]. This process requires an accurate impression or intraoral scan of the neonate’s maxilla to document cleft morphology and fabricate individualized orthopedic appliances.

Traditionally, conventional impressions have been considered a reliable standard for neonatal maxillary registration [5]. However, impression-taking in neonates carries significant risks, including airway obstruction, aspiration or ingestion of impression material, and the potential dislodgement of impression trays or material fragments [6]. Additional complications may include reduced oxygen saturation during the procedure [7, 8] and compromised impression accuracy due to premaxillary mobility, delicate mucosal tissues, and impaired orbicularis oris function in infants with CLP [9]. These challenges, combined with the considerable technical sensitivity of the procedure and the associated stress for clinicians, infants, and caregivers [10], underscore the need for alternative approaches.

In recent years, the digitalization of dentistry has introduced intraoral scanners (IOS) as a promising modality for neonatal maxillary data acquisition [11–13]. IOS systems have been reported to reduce procedural risks and to eliminate the need for impression materials, and they allow for streamlined digital workflows including virtual planning, reproducible data collection, and long-term storage. Early feasibility studies and case reports have demonstrated successful IOS application in neonates with craniofacial anomalies, including CLP [14–22].

However, several important challenges remain. Deep or narrow cleft extensions, steep palatal walls, and posterior anatomical constraints can reduce optical access and increase stitching errors, particularly in complex bilateral clefts [23]. Scanner performance depends on hardware, software and clinical-workflow parameters; as a result, reported trueness and reproducibility vary significantly among intraoral scanner systems [23–25]. These differences are driven by scanner-specific technological variables such as the light-projection or image-acquisition method, optical depth of field, scan-tip (or scan-body) geometry, and the software’s post-processing algorithms [26]. Moreover, edentulous arches present unique challenges: the absence of teeth reduces the number of distinct anatomical landmarks required for robust surface matching, thereby reducing overall scan trueness [27, 28].

At the time this study was conducted, the evidence base on intraoral scanning of cleft-affected neonatal maxillae, particularly comprehensive comparative analyses across multiple scanner types, was limited. Although recent publications have begun to address this gap, few studies directly compare multiple IOS systems under standardized conditions across models representing progressively increasing cleft complexity [24, 29]. The present investigation contributes valuable experimental data toward establishing reliable digital workflows for early cleft care. The aim of this study was to evaluate and compare the precision and trueness of five commonly used intraoral scanners using a standardized landmark-based methodology complemented by full-surface deviation analysis applied to neonatal maxillary models with different cleft types.

## Materials and methods

### Study models

Three anonymized neonatal maxillary plaster casts were selected from the cleft model databank of the University Clinic of Cologne. The study was performed in alignment with the principles outlined in the Declaration of Helsinki in 2013 and was approved by the Ethics Committee of the University Hospital of Cologne (#21-1131_3).

**Fig. 1:**
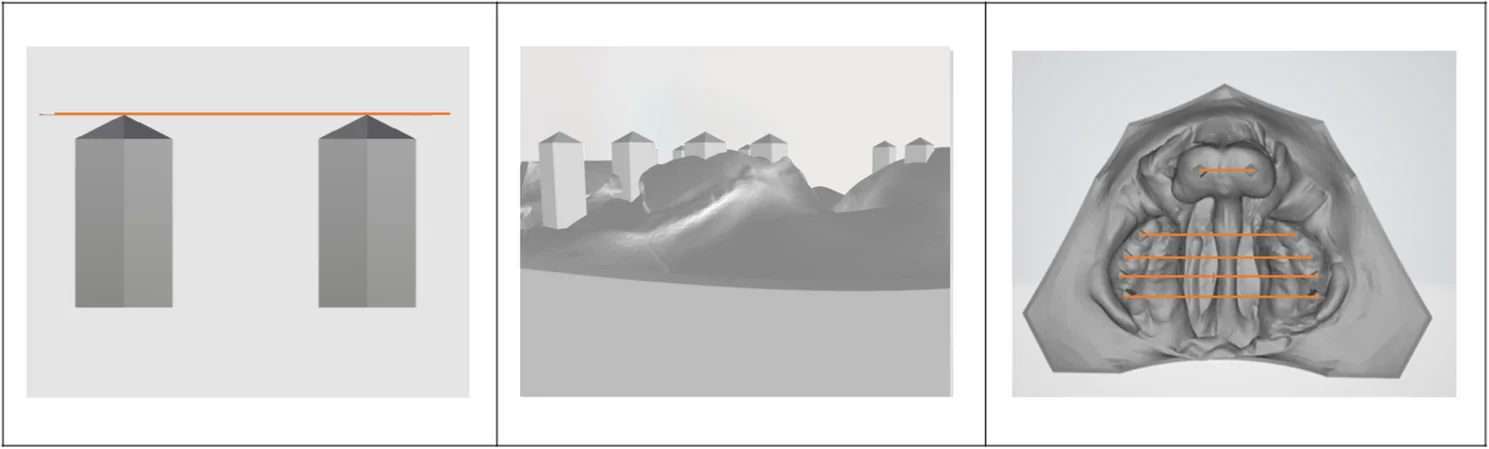
Close-ups of the pyramidal measurement landmarks (1) across the alveolar ridges (2) and the five linear transversal distances for the measurement, distance #1 being the most anterior and distance #5 the most posterior within each model

Study models were categorized into three groups: (1) A neonatal edentulous infant maxilla without cleft morphology, (2) a maxilla with a unilateral cleft on the left side and (3) a maxilla with a bilateral cleft. Each plaster model was digitized using an OrthoX^®^ model scanner (Dentaurum, Ispringen, Germany). STL datasets were processed in ivoris analyze^®^ (Computer konkret, Falkenstein, Germany) and exported for further geometric modelling. In Rhinoceros 3D^®^ (Robert McNeel & Associates, Seattle, USA), ten pyramidal measurement landmarks were digitally constructed and positioned consistently along the alveolar ridge on a standardized horizontal plane (Fig. 1). Five transversal linear distances between predefined landmark pairs were established as the digital baseline reference dataset.

**Fig. 2:**
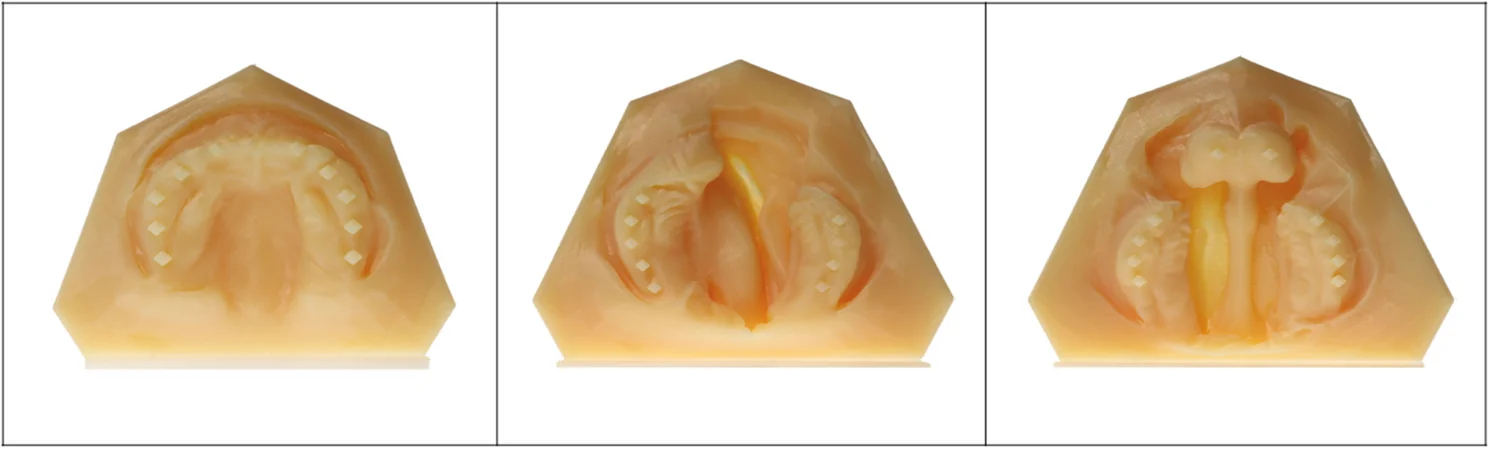
3D-printed models (1) without cleft morphology, (2) with a unilateral cleft on the left side and (3) with a complex bilateral cleft with pyramidal measurement landmarks

Then, the digital master models were printed in a dental laboratory (Implantec, Amstetten, Germany) using a Formlabs Form 2^®^ SLA printer (Formlabs^®^, Somerville, USA) with Dental Model Resin at 25 μm layer resolution (Fig. 2).

After printing, all transversal vertex-to-vertex distances were verified against the digital master using a calibrated digital caliper (accuracy ± 0.01 mm). A contact-based caliper was chosen deliberately, as its measurement error is independent of optical scanning; verifying the printed reference with a second optical scanner would have reintroduced scanner-specific error into the reference standard, the very quantity under investigation. All measured distances matched the digital master within the resolution of the caliper (± 0.01 mm), confirming faithful geometric reproduction of the reference models.

### Scanning procedure

Each printed reference model was scanned ten times using the following five intraoral scanners (IOS):

1. Primescan^®^ (Dentsply Sirona; Wals, Austria)
2. CS3600^®^ (Carestream Health; Rochester, USA)
3. Trios 3^®^ (3Shape A/S; Copenhagen, Denmark)
4. iTero Element 2^®^ (Align Technology; San Jose, USA)
5. Medit i500^®^ (Medit Corp; Seoul, South Korea)

All scans were performed by the same calibrated operator under controlled laboratory conditions. After each set of ten scans, models were re-measured with the same calibrated caliper to rule out geometric deformation caused by repeated handling or environmental influences. No dimensional changes were observed over the course of scanning.

For readability, registered trademark symbols (^®^) are given at first mention only and omitted thereafter.

### Measurement workflow: Landmark-based and full-surface trueness analyses

All IOS-derived STL files were imported into Rhinoceros 3D^®^. Five transversal distances between the ten predefined vertices were recorded for each scan to quantify linear deviations relative to the digital baseline.

For full-surface comparison, STL files were processed in Geomagic Control 3D^®^ (3D Systems, USA) using a standardized two-step alignment protocol:

1. Manual pre-alignment using four corresponding landmark points
2. Best-fit alignment using the iterative closest point algorithm

Each alignment was repeated twice to ensure methodological reproducibility. For every scan, a 3D deviation color map and root mean square (RMS) trueness values as well as in-tolerance percentage (IO%) were generated. IO% denoted the proportion of the surface falling within a ± 0.1 mm tolerance band. The basal socket region used during 3D printing was excluded from analysis to focus exclusively on clinically relevant maxillary structures. Because each superimposition required a two-step alignment performed in duplicate, full-surface deviation analysis was restricted to five randomly selected scans per scanner and model (*n* = 5 per group; 75 datasets in total). The subset was drawn prior to superimposition and therefore independently of any deviation values. The above-mentioned landmark-based analysis, which constitutes the primary outcome of the study, was performed on all ten scans per scanner and model.

**Fig. 3:**
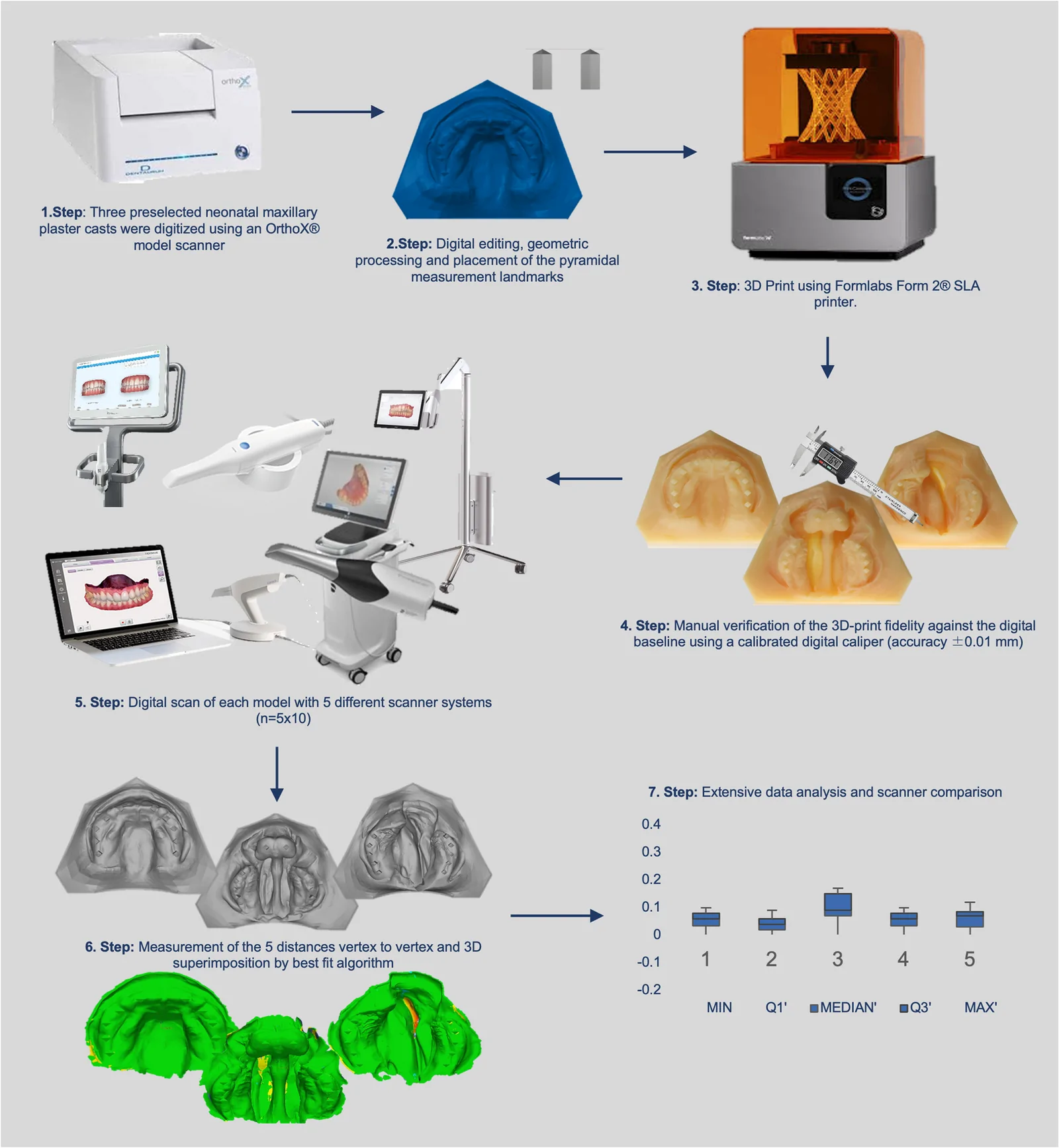
Flow chart of experimental design

Figure 3 illustrates the flow of the experimental design.

### Statistical analysis

Reproducibility of repeated scans (intra-scanner reliability, reflecting precision) and the consistency of repeated measurements (intra-rater reliability) were assessed using Intraclass Correlation Coefficients (ICC).

All linear distance values and trueness metrics were imported into SPSS^®^ (IBM Corp., Armonk, NY) for statistical evaluation. Descriptive statistics (mean, median, standard deviation, minimum and maximum) were calculated for all variables, and boxplots were generated for data visualization. Additionally, for full-surface deviation analysis, RMS deviation, standard deviation, minimum and maximum deviation as well as IO% were computed for each scan. Normality was assessed using the Shapiro–Wilk test. As the assumption of normality was not met for all variables, non-parametric procedures were applied: group comparisons were performed using Kruskal–Wallis and Mann–Whitney U tests at a significance level of *p* < 0.05.

## Results

Intra-rater reliability, reflecting the consistency of repeated landmark identification, was excellent for all scanners and structures, with intraclass correlation coefficients (ICC) between 0.999 and 1.000 and narrow 95% confidence intervals (0.998–1.000). Intra-scanner reliability, reflecting the repeatability of repeated scans, was highest for Primescan (ICC = 1.000), followed by Trios, Carestream and iTero, and lowest for Medit (ICC = 0.832). Together these confirm highly consistent landmark identification and robust scan repeatability across increasing anatomical complexity.

Within this distribution, the spread across the ten repeated scans reflects precision, whereas trueness is given by the mean signed deviations reported (in line with ISO 5725, Tables 1, 2 and 3).

**Table 1:** Mean signed deviations (Δ), standard deviations (SD), minimum (min) and maximum (max) for all five distances (1 being the most anterior and 5 the most posterior distance) and all five IOS for model 1 (no cleft); statistical significance at 5% marked in bold

		1	2	3	4	5	Mean deviation across distances
Primescan	*n* = 10						
	mean	0.0170	0.0250	0.0050	0.0160	0.0100	0.0146
	SD	0.0340	0.0276	0.0613	0.0313	0.0403	0.0229
	min	-0.04	-0.02	-0.09	-0.04	-0.05	-0.01
	max	0.06	0.07	0.08	0.06	0.07	0.05
Trios	*n* = 10						
	mean	-0.0070	0.0040	0.0230	0.0050	0.0060	0.0062
	SD	0.0283	0.0369	0.0263	0.0412	0.0320	0.0110
	min	-0.04	-0.06	-0.01	-0.07	-0.05	0.00
	max	0.05	0.08	0.07	0.06	0.05	0.03
iTero	*n* = 10						
	mean	-0.0020	0.0160	-0.0310	0.0110	0.0190	0.0026
	SD	0.0473	0.0502	0.0748	0.0528	0.0632	0.0250
	min	-0.11	-0.08	-0.15	-0.09	-0.12	-0.04
	max	0.05	0.08	0.12	0.10	0.09	0.04
Carestream	*n* = 10						
	mean	-0.0280	-0.0210	-0.0530	-0.0180	-0.0170	-0.0274
	SD	0.0385	0.0489	0.0497	0.0643	0.0715	0.0295
	min	-0.10	-0.08	-0.11	-0.10	-0.11	-0.07
	max	0.04	0.08	0.03	0.11	0.08	0.01
Medit	*n* = 10						
	mean	0.0120	0.0440	-0.0100	-0.0260	-0.0290	-0.0018
	SD	0.0239	0.0448	0.0521	0.0813	0.0624	0.0279
	min	-0.03	-0.05	-0.08	-0.16	-0.13	-0.04
	max	0.05	0.09	0.11	0.06	0.07	0.04
**Kruskal-Wallis H (df)**		9.870 (4)	11.155 (4)	11.395 (4)	3.033 (4)	4.344 (4)	9.584
**p- value**		**0.043**	**0.025**	**0.022**	0.552	0.361	**0.048**

**Table 2:** Mean signed deviations (Δ), standard deviations (SD), minimum (min) and maximum (max) for all five distances (1 being the most anterior and 5 the most posterior distance) and all five IOS for model 2 (unilateral cleft); statistical significance at 5% marked in bold

		1	2	3	4	5	Mean deviation across distances
Primescan	*n* = 10						
	mean	0.0690	0.0590	0.0350	0.0740	0.0260	0.0526
	SD	0.0493	0.0410	0.0515	0.0406	0.0768	0.0396
	min	-0.01	-0.01	-0.07	0.00	-0.10	0.00
	max	0.15	0.12	0.10	0.15	0.17	0.11
Trios	*n* = 10						
	mean	0.1150	0.0970	0.0650	0.0760	0.0870	0.0880
	SD	0.0363	0.0668	0.0606	0.0538	0.0624	0.0406
	min	0.07	-0.02	-0.02	-0.04	-0.03	0.01
	max	0.17	0.18	0.13	0.13	0.18	0.14
iTero	*n* = 10						
	mean	-0.0410	0.0130	-0.0260	0.0160	0.0120	-0.0052
	SD	0.0530	0.0696	0.0589	0.0807	0.0496	0.0324
	min	-0.12	-0.12	-0.11	-0.07	-0.07	-0.05
	max	0.05	0.12	0.08	0.16	0.07	0.05
Carestream	*n* = 10						
	mean	-0.0240	-0.0530	-0.0730	-0.0470	-0.0310	-0.0456
	SD	0.0822	0.0730	0.0788	0.0566	0.0888	0.0610
	min	-0.14	-0.18	-0.19	-0.13	-0.18	-0.14
	max	0.14	0.05	0.03	0.04	0.12	0.03
Medit	*n* = 10						
	mean	-0.1230	-0.0890	-0.0710	-0.0250	-0.0330	-0.0682
	SD	0.0942	0.1161	0.1088	0.1006	0.0670	0.0816
	min	-0.27	-0.22	-0.22	-0.16	-0.14	-0.19
	max	0.00	0.11	0.10	0.13	0.07	0.04
**Kruskal-Wallis H (df 4)**		32.819	16.774	18.917	18.311	15.486	23.103
**p- value**		**< 0.001**	**0.002**	**< 0.001**	**0.001**	**0.004**	**< 0.001**

**Table 3:** Mean signed deviations (Δ), standard deviations (SD), minimum (min) and maximum (max) for all five distances (1 being the most anterior and 5 the most posterior distance) and all five IOS for model 3 (bilateral cleft); statistical significance at 5% marked in bold

		1	2	3	4	5	Mean deviation across distances
Primescan	*n* = 10						
	mean	0.0240	0.0120	-0.0090	0.0050	-0.0360	-0.0008
	SD	0.0327	0.0399	0.0456	0.0264	0.0409	0.0121
	min	-0.03	-0.06	-0.09	-0.04	-0.10	-0.02
	max	0.07	0.07	0.06	0.06	0.02	0.02
Trios	*n* = 10						
	mean	0.0320	0.0160	-0.0010	0.0030	-0.0340	0.0032
	SD	0.0333	0.0384	0.0536	0.0267	0.0443	0.0177
	min	-0.03	-0.06	-0.09	-0.04	-0.10	-0.02
	max	0.07	0.07	0.08	0.06	0.04	0.04
iTero	*n* = 10						
	mean	0.0300	0.0120	-0.0590	-0.1400	-0.0840	-0.0482
	SD	0.0460	0.0864	0.0731	0.1020	0.1115	0.0591
	min	-0.08	-0.13	-0.20	-0.25	− 0.035	-0.15
	max	0.09	0.14	0.05	0.03	0.01	0.04
Carestream	*n* = 10						
	mean	0.0200	-0.0240	-0.0140	0.0170	-0.0420	-0.0086
	SD	0.0323	0.0737	0.0786	0.0591	0.0834	0.0501
	min	-0.02	-0.17	-0.13	-0.09	-0.21	-0.11
	max	0.08	0.08	0.11	0.09	0.06	0.05
Medit	*n* = 10						
	mean	0.0010	-0.1830	-0.1670	-0.1990	-0.1870	-0.1470
	SD	0.0218	0.0663	0.0882	0.0848	0.0483	0.0439
	min	-0.03	-0.29	-0.36	-0.36	-0.27	-0.25
	max	0.05	-0.06	-0.06	-0.08	-0.13	-0.09
**Kruskal-Wallis H (df)**		7.577	23.104	21.245	27.843	19.880	25.442
**p- value**		0.108	**< 0.001**	**< 0.001**	**< 0.001**	**< 0.001**	**< 0.001**

**Fig. 4:**
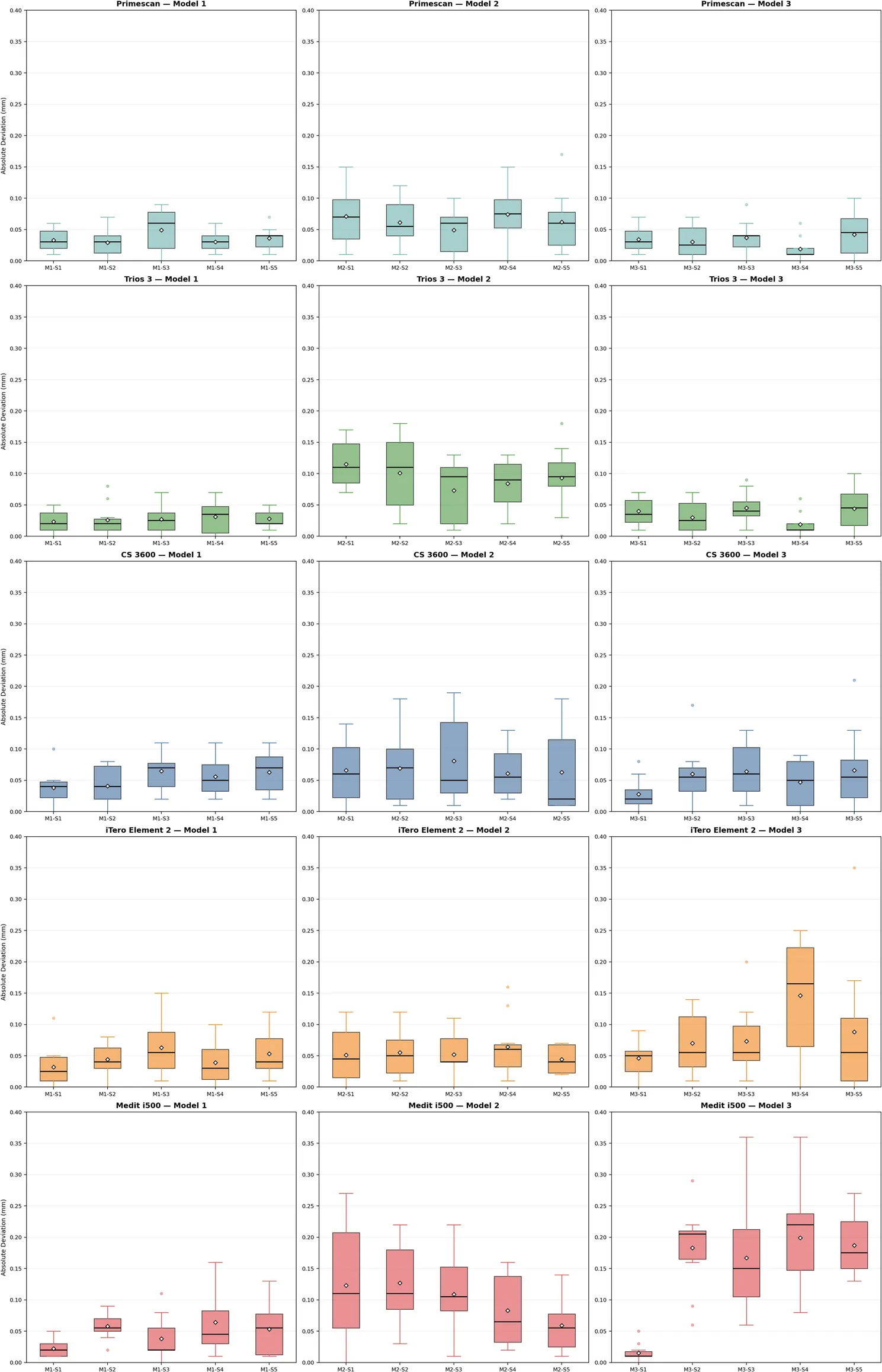
Absolute deviation from reference [mm] per IOS and model

The distribution of absolute linear deviations from the reference (|Δ|; Fig. 4) showed consistent scanner-specific patterns. On the non-cleft model, all scanners produced narrow deviation ranges with median |Δ| values typically below 0.05 mm. Primescan and Trios showed the smallest variability, while Carestream and Medit demonstrated slightly wider spreads. Precision decreased modestly on the unilateral cleft model, with more isolated outliers - particularly for Trios - although most median deviations remained below 0.10 mm. The bilateral cleft model produced a notable spread of deviations. Overall, Primescan exhibited the most stable precision across all models, followed by Trios and Carestream, whereas iTero and Medit showed increased variability with cleft complexity.

**Fig. 5:**
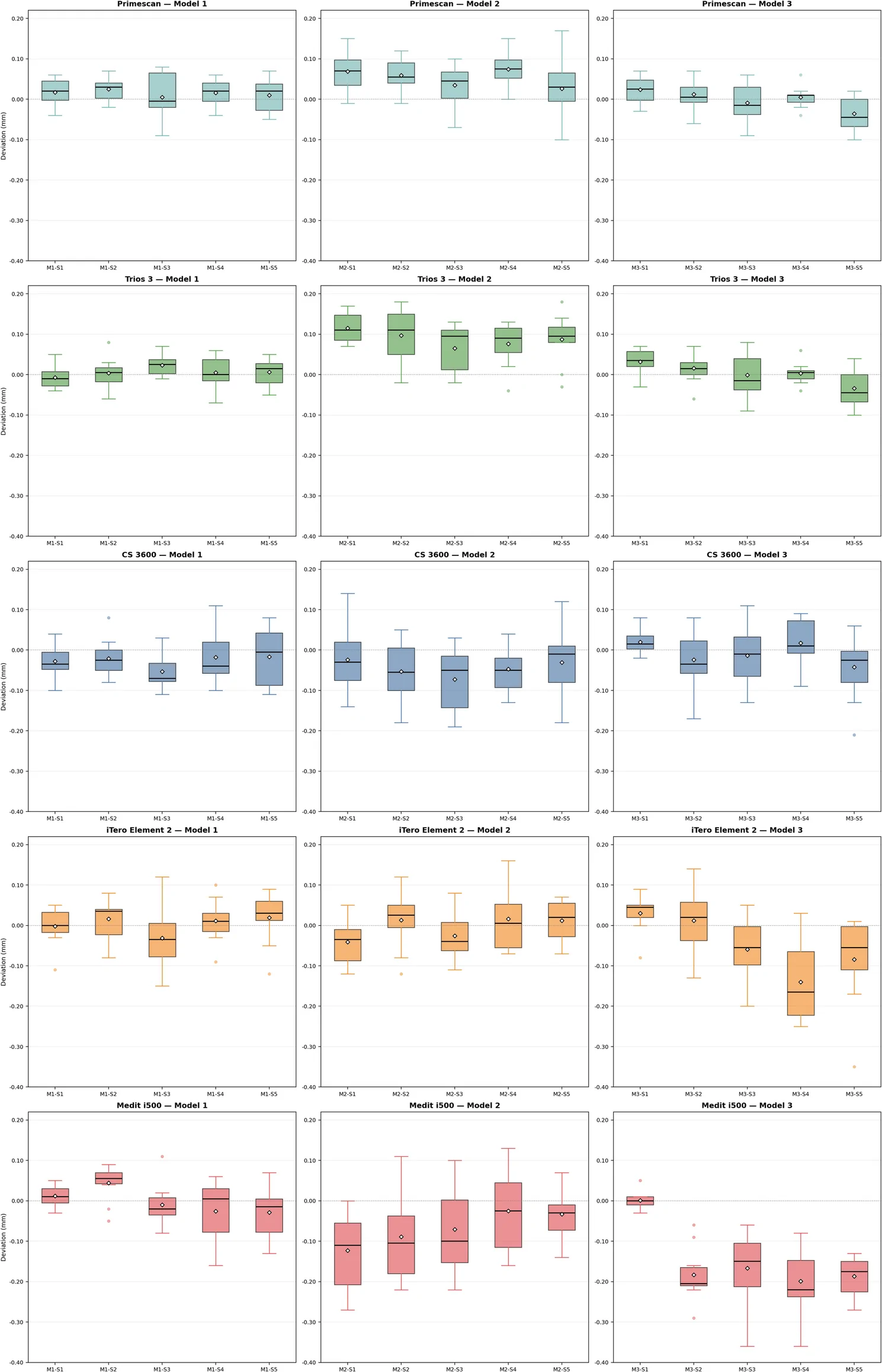
Signed deviation from reference [mm] per IOS and model

Analysis of signed deviations (Δ) revealed minimal systematic error in the non-cleft model, with mean deviations ranging from − 0.0274 mm for Carestream to + 0.0146 mm for Primescan and negligible values for Trios, iTero and Medit. A small, marginally significant scanner difference was detected (*p* = 0.048; Table 1; Fig. 5). In the unilateral cleft model, scanner-specific bias became more pronounced: Primescan and Trios showed slight positive deviations, iTero remained near zero, and Carestream and Medit demonstrated systematic underestimation. Differences between scanners were significant (*p* < 0.001; Table 2; Fig. 5). In the bilateral cleft model, Primescan and Trios again remained essentially unbiased, while Carestream and iTero showed moderate underestimation and Medit demonstrated a clear and consistent negative bias (-0.147 mm). Scanner differences were significant (*p* < 0.001; Table 3; Fig. 5), with Medit differing from all other devices across most structures.

Full-surface trueness (Table 4) differed significantly between scanners in all three models (RMS: *p* < 0.001, *p* = 0.004 and *p* = 0.002; IO%: *p* < 0.001, *p* = 0.008 and *p* < 0.001 for models 1–3 respectively). Four of the five devices achieved mean RMS deviations below 0.07 mm in every model. iTero was the exception: its mean RMS rose from 0.087 mm in the non-cleft model (IO% 97.53%) to 0.179 mm in the unilateral (IO% 95.26%) and 0.224 mm in the bilateral cleft model (IO% 94.19%). On the non-cleft model, Primescan achieved the highest trueness (mean RMS 0.030 mm; IO% 99.81%), followed by Trios and Medit (mean RMS for both 0.037 mm; IO% 98.95% and 98.57% respectively) and Carestream (mean RMS 0.042 mm; IO% 97.25%). On the unilateral cleft model, mean RMS increased for all five devices; this model also produced the lowest in-tolerance percentage of the study for Primescan, Trios and Medit, with Medit falling to 93.21%. On the bilateral cleft model, mean RMS was lowest for Medit (0.051 mm), followed by Trios (0.059 mm), Carestream (0.060 mm) Primescan (0.069 mm) and iTero (0.224 mm).

**Table 4:**
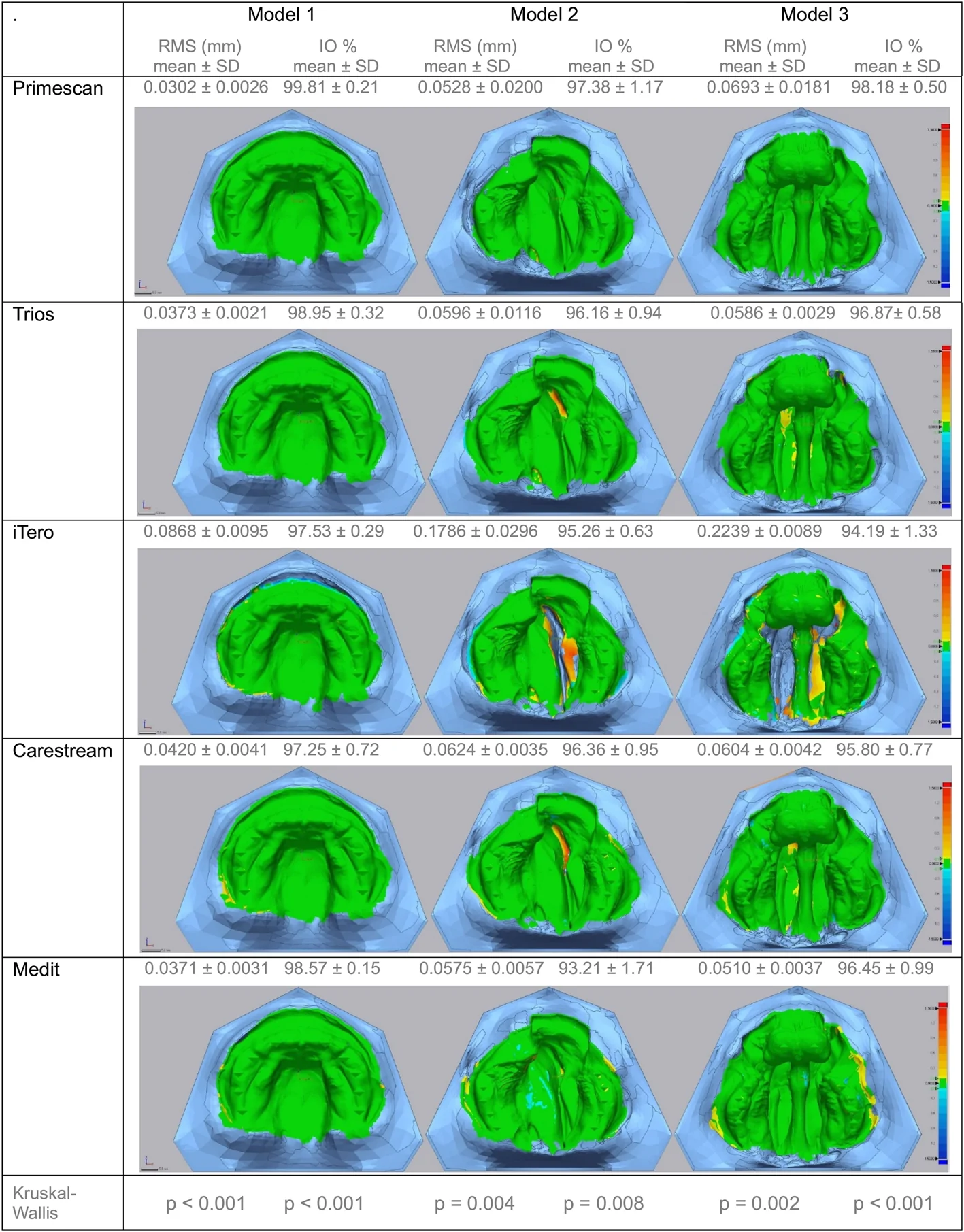
Full-surface trueness analysis (Geomagic Control 3D^®^); deviation color map (green areas representing deviations within ± 0.1 mm); mean root mean square (RMS) deviation from digital master model and mean in-tolerance percentage (IO %), SD = standard deviation; statistical significance at 5%

The two analyses (linear landmark deviations and full-surface trueness) did not rank the devices identically. Medit showed the largest linear bias in the bilateral cleft model but among the lowest full-surface RMS deviations, whereas iTero showed moderate linear deviations together with the poorest surface reconstruction.

## Discussion

This study evaluated the trueness and precision of five intraoral scanners (IOS) in the digital registration of neonatal maxillary models representing increasing anatomical complexity: a neonatal edentulous ridge, a unilateral cleft, and a bilateral cleft configuration. The objective was to determine whether these scanner systems are capable of accurately capturing different cleft morphologies, to identify regional and between-scanner differences, and to assess how model complexity influences trueness and repeatability. Overall, the results demonstrated that all tested intraoral scanners were capable of digitizing neonatal maxillary models with and without cleft morphology. However, between-scanner differences were statistically significant in both cleft models and in both the landmark-based and the full-surface analysis. The two analyses did not, however, rank the devices identically, which is central to the interpretation developed below.

Anatomical complexity affected all devices, but not as a uniform progression. In the full-surface analysis, the non-cleft control model produced the lowest mean RMS for every scanner, confirming high baseline trueness under non-complex conditions, and RMS increased for all five devices from the non-cleft to the unilateral cleft model. Beyond that point the pattern was no longer monotonic: RMS did not increase further in the bilateral cleft model for Trios, Carestream or Medit, and for three of the five devices the in-tolerance percentage was lowest in the unilateral rather than the bilateral cleft model. The landmark-based analysis showed the same device dependence. Only iTero and Medit deviated progressively more with each step in complexity, reaching mean deviations of − 0.048 mm and − 0.147 mm respectively in the bilateral cleft model, whereas Primescan, Trios and Carestream showed their largest mean deviations in the unilateral cleft model and returned to near-baseline values in the bilateral cleft model. Deeper fissures, pronounced undercuts and restricted optical access therefore clearly challenge accurate capture, but the level of morphological complexity alone does not predict the magnitude of error; the interaction between a given device and a given morphology does. These findings align with the observations of Chatborirak et al. [29] and Unnikrishnan et al. [24, 30], who reported a progressive decline in scan accuracy with increasing morphological irregularity and reduced optical accessibility.

The anterior-to-posterior landmark layout was chosen a priori to allow positional effects to be examined. No monotonic anterior–posterior gradient was observed, and no consistent positional pattern was present across scanners and models. As no statistical test for position as a factor was performed, the following observations are descriptive only. In the bilateral cleft model, the two devices with the lowest overall trueness deviated substantially outside the most anterior distance: iTero showed its largest deviations at the two posterior distances (up to − 0.140 mm), while Medit deviated by − 0.167 to − 0.199 mm at every distance except the most anterior one. In contrast, Primescan and Trios remained within ± 0.04 mm at every position in this model. This is best interpreted, qualitatively, as a scanner- and morphology-dependent loss of accuracy in regions of restricted optical access within the deep bilateral cleft, rather than as a generalized positional gradient. Reduced trueness in posterior palatal vaults has been reported for several IOS types by Ferrari et al. [23] and ElNaghy et al. [31], who attributed it to restricted light penetration and increased reflection noise; the present data are consistent with such a mechanism operating in specific devices and regions rather than across the arch as a whole.

Statistically significant performance differences between scanners were evident in the landmark analysis in both cleft models. Primescan and Trios demonstrated the highest trueness and the lowest intra-scanner variability in the non-cleft and bilateral cleft models; in the unilateral cleft model both showed a positive mean bias, most pronounced for Trios (+ 0.088 mm). In this analysis iTero and Carestream remained within commonly cited tolerance limits but exhibited greater variability in the bilateral cleft model, whereas Medit showed the largest mean deviations and the highest variability, especially in posterior cleft segments, reaching − 0.147 mm in the bilateral cleft model.

Surface-based deviation analysis did not simply reproduce the landmark findings. More importantly than the differing complexity patterns described above, the two analyses rank the devices differently: Medit produced the largest linear bias of any device in the bilateral cleft model (− 0.147 mm) yet the lowest full-surface RMS in that same model (0.051 mm), whereas iTero produced moderate linear deviations alongside full-surface RMS values more than three times those of any other device. A plausible explanation is that the two metrics are sensitive to different failure modes. The transversal landmark distances capture systematic dimensional bias across the arch, whereas RMS is dominated by localized reconstruction failure in regions of restricted optical access. A device may therefore reproduce individual palatal surfaces faithfully while under-registering overall arch width, or the reverse. This argues for interpreting the two analyses in parallel rather than ranking scanners on either metric alone.

The differences observed in both analyses cannot be attributed to any single technological component, as complete device systems rather than isolated imaging technologies were compared. One plausible contributing factor is the underlying imaging approach: confocal and hybrid-projection systems such as Trios and Primescan may capture deeper structures in focus and provide improved fidelity in undercut or complex regions, whereas the structured-light approach used in Carestream and Medit may be more sensitive to shadowing in confined areas, potentially yielding local under-registration, scatter artefacts or stitching inconsistencies. Such a tendency would be consistent with previous reports, including Gehrke et al. [26], who highlighted that structured-light systems show greater susceptibility to depth-related errors in steep or narrow anatomical segments. The present data, however, do not support this explanation uniformly. As reported above, iTero Element 2 also employs confocal imaging, yet produced by far the poorest full-surface reconstruction of the five devices, with the lowest in-tolerance percentage in the bilateral cleft model (94.19%) and the highest mean RMS in the non-cleft model (0.0868 ± 0.0095 mm). The imaging principle alone therefore cannot account for the observed ranking. Additional device-specific factors, including scanning software, scanning strategy, device configuration, proprietary post-processing algorithms, scan-tip geometry and frame-capture frequency, may contribute equally and cannot be disentangled in the present comparison of whole systems. These differences are therefore attributed at device rather than technology level.

Regional, scanner-specific weaknesses were also identifiable qualitatively and are consistent with this device-level interpretation. iTero showed increased variability in the posterior palatal segments and, in several scans, displayed a tendency to partially smooth or erase deep cleft contours during automatic surface reconstruction, which made subsequent registration of the bilateral cleft model markedly more challenging and offers a plausible source of its elevated RMS values. Carestream demonstrated greater error accumulation along steep lateral cleft margins in the unilateral model, consistent with structured-light systems being more sensitive to shadowing and undercuts. Primescan maintained largely uniform trueness across regions, with only slight reductions in accuracy near the alveolar ridge junction in the bilateral cleft model. These region-dependent patterns align with earlier findings by Keil et al. [18], who reported that scanner ergonomics, acquisition angle, and field-of-view exert strong influence on local accuracy in pediatric maxillary scanning. In the cleft models, between approximately 2% and 7% of the scanned surface fell outside the ± 0.1 mm tolerance band for every device, with the out-of-tolerance regions concentrated in the deep cleft and posterior palatal areas visible in the deviation colour maps. Such localized errors are averaged out in linear landmark measurements and are therefore only apparent in the surface-based analysis.

Intra-scanner reliability ranged from ICC 0.832 to 1.000 across all scanners and models. Together with the excellent intra-rater reliability (ICC 0.999–1.000), these findings support the conclusion that most variation arises from scanner systems rather than operator-related factors. They also corroborate previous studies reporting high scan reproducibility under standardized in-vitro conditions [11].

Beyond these relative comparisons (between scanners and across levels of model complexity), the absolute magnitude of the deviations observed in this study must be interpreted in the context of established clinical thresholds. According to practical guidelines from relevant literature, deviations below 0.10 mm (100 μm) are considered clinically acceptable for orthodontic models and presurgical orthopedic appliance fabrication in neonates [18, 29], while thresholds around 0.05 mm (50 μm) are commonly cited for high-precision applications requiring superior trueness [32–34]. All Primescan mean deviations across the three models fell within these limits, although individual measurements in the unilateral cleft model reached 0.17 mm. Trios showed comparably low mean deviations in the non-cleft and bilateral cleft models. iTero and Carestream also remained below the 0.10 mm threshold in mean terms in every model, with Carestream deviating most in the unilateral rather than the bilateral cleft model. Medit was the only device whose mean deviation exceeded this threshold, in the bilateral cleft model (− 0.147 mm). Primescan and Trios were the only two devices that performed consistently well in both analyses, combining near-zero linear bias in the non-cleft and bilateral cleft models with mean RMS below 0.07 mm in every model. For clinical practice, these findings highlight the need for careful scanner selection and scanning strategy in infants with complex cleft anatomy, where even moderate deviations may affect appliance fit or growth documentation, and they indicate that a device should not be selected on the basis of either metric alone.

The present findings complement and extend the growing body of literature supporting digital workflows in neonatal cleft care. By including both unilateral and bilateral cleft models, this study broadens the scope of earlier in-vitro investigations that focused primarily on unilateral defects, and by combining a landmark-based with a full-surface analysis it shows that the two approaches are sensitive to different failure modes. The stronger performance of Primescan and Trios in this sample points to their suitability for complex neonatal morphologies and underscores the need for further refinement of the remaining systems for use in deep and narrow cleft geometries. Digital scanning has been associated with potential procedural advantages such as reduced airway interference, shorter procedure time, improved caregiver comfort and seamless CAD/CAM integration [6–8, 10, 14, 19, 22]; these aspects were not assessed in the present in-vitro study and are noted here only as context. Within the scope of what was tested, these findings support the growing interest in intraoral scanning as a potential alternative to conventional impressions in neonatal cleft care. Considering the rapid advancements in optical design, reconstruction algorithms, and scanner ergonomics, the outlook for digital cleft care is encouraging. The present study is limited to the five IOS systems evaluated. Emerging systems with enhanced depth capture and real-time error correction are likely to further improve accuracy and expand the clinical potential of fully digital workflows in neonatal cleft management.

## Limitations

This was an in-vitro investigation on 3D-printed models and therefore characterizes technical accuracy under controlled laboratory conditions rather than clinical performance in neonates; factors such as patient movement, soft tissue, saliva, limited mouth opening and the completeness of intraoral capture in infancy were not assessed. Deviations measured against the digital master could in principle include a residual 3D-printing component; however, print fidelity was independently verified by digital caliper (± 0.01 mm) post-print and mid-campaign for the transversal landmark distances. Because all five scanners captured the identical printed models, any shared print error cancels from the between-scanner and across-model comparisons that constitute the main findings; this argument, rather than the caliper verification, underwrites the full-surface analysis, where deep cleft and undercut regions are not accessible to a contact instrument. The full-surface analysis was based on a random subset of five of the ten scans acquired per scanner and model. Although the subset was fixed before superimposition and could not be influenced by the resulting deviation values, the smaller group size reduced the precision of the surface estimates relative to the landmark-based analysis.

Scanning was performed by a single calibrated operator; intra-rater reliability was excellent (ICC 0.999–1.000), indicating a negligible operator contribution, though inter-operator variability was not examined. Each model was scanned ten times per device, and no conventional-impression comparator arm was included. These constraints should be considered when interpreting the results.

## Conclusion

This study demonstrates that intraoral scanning can digitize neonatal maxillary anatomy with high technical accuracy under in-vitro conditions across edentulous, unilateral cleft, and bilateral cleft configurations. Although accuracy depended on the interaction between device and morphology rather than on the level of anatomical complexity alone, all IOS were fundamentally able to capture cleft morphology, with Primescan and Trios achieving the highest trueness and reproducibility in this sample.

The remaining devices showed greater variability, especially in regions of restricted optical access, but remained adequate for less intricate anatomy. Clinical feasibility in neonates was not assessed here and remains to be established through in-vivo investigation in this vulnerable patient group.

## Supplementary Information

Supplementary Material 1.

## Data Availability

The datasets generated and/or analyzed during the current study are not publicly available due to confidentiality restrictions but are available from the corresponding author on reasonable request.
