# Supplementary material for: Evaluation of precision and trueness of intraoral scanners for newborns with cleft lip and palate: 3D analysis of digital models
Source: Head Face Med. 2026 Sep 9;22:75. doi: 10.1186/s13005-026-00650-w (PMC13560194; doi:10.1186/s13005-026-00650-w)
Supplement: Supplementary file 1 [file 13005_2026_650_MOESM1_ESM.doc]

Prof. Dr. Thomas Stamm

Editor-in-Chief

*Head & Face Medicine*	01.08.2026

Dear Professor Dr. Stamm,

please find enclosed our revised manuscript, *"Evaluation of precision and trueness of intraoral scanners for newborns with cleft lip and palate: 3D analysis of digital models"* (Manuscript ID 4adff653-7753-437d-b454-1a39d39b7190), submitted for consideration in Head & Face Medicine.

We are grateful to both reviewers for their careful and constructive assessment. We have revised the manuscript in full accordance with their comments. In brief, we have made the terminology consistent with ISO 5725 throughout, replaced the claim of an anterior–posterior accuracy gradient with a narrower descriptive statement supported by our data, acknowledged the 3D-printing component of the reference standard, modified table 4 through reporting the full-surface results, and tempered the conclusions so that they describe technical accuracy under in-vitro conditions rather than clinical feasibility in neonates. The Discussion has been restructured accordingly. A point-by-point response to all comments accompanies this submission.

The manuscript has still not been published elsewhere and is still not under consideration by another journal. All authors have read and approved the revised version and take full responsibility for its content.

Yours sincerely,

PD Dr. Isabelle Graf

Department of Orthodontics ▪ Faculty of Medicine and University Hospital Cologne

Kerpener Str. 32 ▪ 50931 Cologne, Germany

Tel: +49-(0)221-478-98520 ▪ Fax: + 49-(0)221-478-96754 ▪ E-mail: [isabelle.graf@uk-koeln.de](mailto:isabelle.graf@uk-koeln.de)
